# Positive influence of short message service and voice call interventions on adherence and health outcomes in case of chronic disease care: a systematic review

**DOI:** 10.1186/s12911-016-0286-3

**Published:** 2016-04-22

**Authors:** F. Yasmin, B. Banu, S. M. Zakir, R. Sauerborn, L. Ali, A. Souares

**Affiliations:** Institute of Public Health, University of Heidelberg, INF 324, Heidelberg, 69120 Germany; Bangladesh University of Health Sciences, 125/1, Darus Salam, Mirpur-1, Dhaka, 1216 Bangladesh

**Keywords:** M-Health, Mobile phone, Short message system (SMS), Adherence, Health outcomes, Chronic disease

## Abstract

**Background:**

Chronic diseases have emerged as a serious threat for health, as well as for global development. They endenger considerably increased health care costs and diminish the productivity of the adult population group and, therefore, create a burden on health, as well as on the global economy. As the management of chronic diseases involves long-term care, often lifelong patient adherence is the key for better health outcomes. We carried out a systematic literature review on the impact of mobile health interventions -mobile phone texts and/or voice messages- in high, middle and low income countries to ascertain the impact on patients’ adherence to medical advice, as well as the impact on health outcomes in cases of chronic diseases.

**Methods:**

The review identified fourteen related studies following the defined inclusion and exclusion criteria, in PubMed, Cochrane Library, the Library of Congress, and Web Sciences. All the interventions were critically analysed according to the study design, sample size, duration, tools used, and the statistical methods used for analysing the primary data. Impacts of the different interventions on outcomes of interest were also analysed.

**Results:**

The findings showed evidence of improved adherence, as well as health outcomes in disease management, using mobile Short Message Systems and/or Voice Calls. Significant improvement has been found on adherence with taking medicine, following diet and physical activity advice, as well as improvement in clinical parameters like HbA1c, blood glucose, blood cholesterol and control of blood pressure and asthma.

**Conclusions:**

Though studies showed positive impacts on adherence and health outcomes, three caveats should be considered, (i) there was no clear understanding of the processes through which interventions worked; (ii) none of the studies showed cost data for the m-health interventions and (iii) only short term impacts were captured, it remains unclear whether the effects are sustained. More research is needed in these three areas before drawing concrete conclusions and making suggestions to policy makers for further decision and implementation.

## Background

Chronic diseases, both communicable (CD) and non-communicable (NCD), have emerged as serious challenges for health, as well as global development. They have substantially increased health care costs, limited productivity, especially in low and middle income countries (LMICs), and added to the existing burden of poverty on countries’ economy. Among chronic communicable diseases (CDs), HIV/AIDS is the most common cause of morbidity and mortality worldwide. According to World Health Organization (WHO), approximately 35.0 million people were living with HIV at the end of 2013, with 2.1 million people globally becoming newly infected in 2013. The burden of the epidemic varies considerably between countries and regions, with the majority, 2.9 million, being in low-and middle income countries [2]. Sub-Saharan Africa is the most affected region, with 24.7 million people living with HIV in 2013, and accounting for almost 70 % of the global total of new HIV infections [29, 40].

In 2010, the WHO Global Status Report on NCDs showed that NCDs are the biggest cause of death worldwide. NCDs are estimated to contribute almost 36 million (80 %) global annual deaths, of which 29 million occur in LMICs. NCD deaths are projected to increase by 15 % globally between 2010 and 2020 (44 million deaths). The greatest increases, by 20 %, will be in Africa, the Eastern Mediterranean and South East Asia. WHO does not estimate an increase in the European Region. Lack of accessibility, inequality and inequity in health service provision are the main barriers to provide services for chronic disease care, mainly in LMICs. Follow-up is mostly sporadic, while adherence to treatment, life style changes, and self management is overlooked, community services are ignored, prevention is not emphasized and referral linkage is not well established and/or not functional [28].

In 2013, global mobile phone penetration rates stood at 96 % worldwide (Information and Communication Technology ICT facts and figures, [18]). Use of mobile technologies in health, as well as advancement in innovative applications to address health priorities, has evolved into a new field of e-health known as m-Health (mobile health). E-health is a term for health care practices supported by electronic/ digital processes and also refers to health care practices using the internet. m-Health is a new field of e-health which involves the use of mobile phone core functions including voice and short messaging services (SMS), as well as more complex functions and specialized applications. For example, bluetooth technology could be linked with health parameter devices (e.g., glucometer) [29]. Interventions may also include a phone platform where patients can call, in case they have questions or emergency. The growing mobile phone networks, with increased accessibility providing the opportunity for more personalized and citizen-centered medical care, have been found to lower the health system barriers, particularly patient access, as well as reduce the health care service cost [19]. m-health is now being used as a tool to achieve a number of other objectives: accessing people irrespective of socio-economic status (SES), increasing patients’ satisfaction with quality health care and improving adherence, which, in the end, benefits patients, providers, and health care systems. However, mobile health care interventions are still new and, to the best of our knowledge, no systematic review has yet been published that analyses evidence on whether the use of mobile phone interventions (SMS or voice message and/or interactive call and emergency call) improves patient adherence or the process of care and health outcomes, without linkage to any other web-based interventions (e-mail, skype etc.). In the context of LMICs with low internet penetration and low smartphone penetration, as well as low literacy rate and individual SES (socio-economic status) it is important to use mobile applications independently of internet access to be able to reach all types of individuals [15, 27].

Therefore, the aim of this review is to gather scientific evidence on the effective uses of mobile phones through SMS and/or voice messages, with or without emergency phone calls and/or interactive calls, to see the effect on patients’ adherence and clinical outcomes, with the objective of advising policy makers on the use and effective implementation of m-Health technologies.

## Methods

### Data sources

PubMed, Cochrane Library, the Library of Congress, and Web Sciences were searched for related articles. The keywords used for the search were – “m-Health”, “mobile communication”, “telemedicine technologies”, “interventions”, “adherence”, “compliance”, “chronic diseases”, “chronic conditions”, “randomized control trial”, “clinical trial”, “experimental design”, “quasi-experimental design”, and “observational study”. The same keywords have been used for all search engines.

### Inclusion and exclusion criteria

The eligibility criteria for study inclusion were (1) patients with chronic diseases (both communicable and non-communicable) living in high, middle or low income countries, (2) mobile health intervention, (3) experimental, quasi- experimental, and observational studies, (4) publications in peer-reviewed journals, and (5) publications written in English. Exclusion criteria were (1) studies focusing on non chronic diseases (2) studies in which a mobile phone was used together with other web based interventions, (3) reviews, policy papers, feasibility studies, grey publications, reports, book chapters, and master/bachelor theses, (4) publications not written in English and (5) publications in non peer-reviewed journals.

### Data extraction

**Fig. 1:**
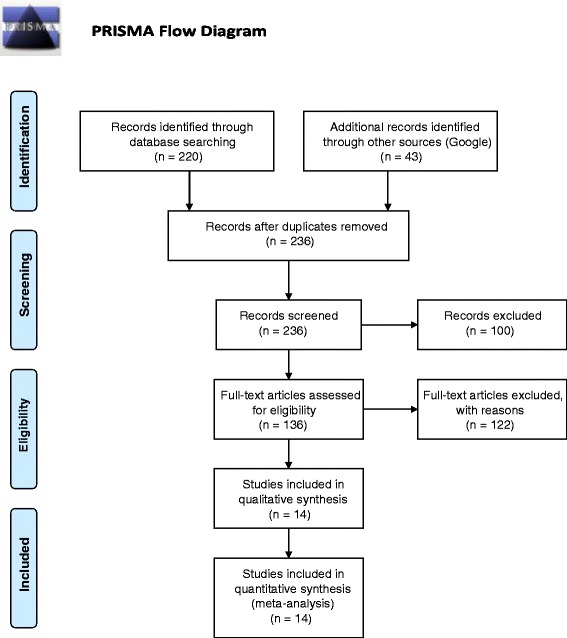
Review process of the articles. Two of the authors independently searched for articles using the same search engines (PubMed, Cochrane Library, the Library of Congress, and Web Sciences) and the same key words (“m-Health”, “mobile communication”, “telemedicine technologies”, “interventions”, “adherence”, “compliance”, “chronic diseases”, “chronic conditions”, “randomized control trial”, “clinical trial”, “experimental design”, “quasi-experimental design”, and “observational study”). All papers published up to May 2014 were considered when the review has been done. All the titles and abstracts has been screened to decide whether should be included in the review paper following the inclusion and exclusion criteria; grey and policy papers, systematic reviews or meta-analysis and articles focusing on non-chronic conditions or diseases were excluded. The articles which includes additional interventions, like web based interventions, together with mobile phone SMS and voice call interventions, were also excluded. Finally, 14 articles met the full inclusion criteria and incorporated in the paper

Two of the authors (FY and BB) independently searched for articles using the same search engines and the same key words list. After the initial individual search, 236 potential articles were identified (FY 164, BB 99, among them 27 articles were common to both reviewers). We considered all papers published up to May 2014 when the review has been done. The first author (FY) has then reviewed all the titles and abstracts to decide whether the full text should be examined. 66 grey and policy papers, 23 systematic reviews or meta-analysis and 11 articles focusing on non-chronic conditions or diseases were excluded. After the primary exclusion, 136 full texts were screened. Finally, 122 articles which includes additional interventions, like web based interventions, together with mobile phone SMS and voice call interventions, were excluded. At the end, 14 articles met the full inclusion criteria. (Fig. 1).

### Data analysis

The reviewed articles were categorized according to clinical areas (Diabetes, HIV/Anti-Retro Viral Therapy, Asthma and Hypertension) and analysed according to the study design, sample size, intervention (process and duration), methodological traits, tool/s used as well as the frequency of use, measurement of outcome, and research location. The researchers carefully checked for the tools used for measuring adherence, both at baseline and end-line of the intervention, for both control and intervention groups. The results of the interventions were analysed according to the clinical areas, as well as across the clinical areas and extracted into a matrix (Tables 1, 2 and 3). The review has been done following the PRISMA.

**Table 1:** Key findings in the clinical area of Diabetes Mellitus Type I and II

Lead Author/Year	Country	Study design	Sample size	Duration	Age group (in Years)	Intervention	Delivery frequency	Measures of outcome	Results – control (C) versus intervention (I)
Ananth Samoth Shetty/2011 [34]	India	RCT	215	12 months	30–65	SMS	Once in 3 days	Adherence to management prescription	Hb1Ac level
									I: 30.8 to 55.1 %
									C: 31.8 % to 48.5 %.
V. L. Franklin/ 2006 [13]	Scotland	RCT	90	12 months	8–18	SMS, emergency hotline	Daily SMS	Self-efficacy, adherence to treatment	I and C using conventional therapy:10.3 ± 1.7 vs. 10.1 ± 1.7 %
									I with intensive insulin therapy and Sweet Talk: 9.2 ± 2.2 %, 95 % CI −1.9, −0.5, *P* < 0.001
									Self-reported adherence: conventional therapy 70.4 ± 20.0, conventional therapy plus Sweet Talk 77.2 ± 16.1, 95 % CI +0.4, +17.4, *P* = 0.042
M. Vervloet/2012 [38]	Netherland	RCT	104	6 months	18–65	SMS reminder	SMS only incase patient didn’t open the medication dispenser	Adherence to oral hypoglycaemic agent	Doses within predefined time windows, within a 1-h window
									I: 50 %
									C:39 %
									within a 4-h window
									I: 81 %
									C: 70 %
Mandana Goodarzi/2012 [16]	Iran	RCT	81	3 months	30+	SMS	4 SMS weekly	Improving laboratory test levels and Knowledge, Attitude, Practice (KAP) and Self Efficacy (SE) of patients	HbA1C level
									C: 7.83 to 7.48 %
									I: 7.91 to 7.02 %.
									LDL level
									C: 99.13 mg/dl to 98.95 mg/dl
									I: 97.88gm/dl to 87.93gm/dl.
									Triglyceride
									C: 173.4 mg/dl to 169.08 mg/dl
									I: 179.72 gm/dl to 160.16gm/dl.
									Knowledge improved 7.97 to 10.83 %, practice 3.72 to 4.93 %, and SE 15.34 to 17.02 % in I I group.

**Table 2:** Key findings in the clinical area of HIV/AIDS (Anti-retro Viral Therapy)

Lead Author/Year	Country	Study design	Sample size	Duration	Age group	Intervention	Delivery frequency	Measures of outcome	Results Control (C) versus Intervention (I)
Cristian Pop-Eleches/2011 [32]	Kenya	RCT	428	12 months	18+	SMS- Short and long	Short- daily, Long- weekly	Adherence to ART	Adherence of at least 90 % during 48 weeks of study in
									I: 53 %
									C: 40 %
Richard T Lester /2010 [25]	Kenya	RCT	538	12 months	18+	SMS	Weekly	Drug adherence, suppression of plasma viral load	Adherence to ART
									I: 61.5 %; C: 49.81 %. Suppressed plasma viral loads I: 60.4; C: 48.3 %.
Rashmi Rodrigues/2012 [33]	India	Quasi-experimental cohort	150	6 months	18+	Interactive voice call and picture message	Weekly	Adherence, Pill count	Adherence at baseline, month 1, month 3, month 6, month 9 and month 12 were 85 %, 94 %, 93 %, 91 %, 95 %, and 94 % respectively.
Setor Kunutsor/2010 [24]	Uganda	Cross-sectional and descriptive	176	7 months	18+	Voice call and SMS	4 weekly	Attendance. Drug refill	Mean adherence before and after intervention was 96.3 % and 98.4 % respectively ((95 % confidence interval).
Dongsheng Huang/2013 [17]	China	RCT	172	3 months	18+	Voice call	2 weekly	Adherence to ART and quality of life (QOL)	CD4 count:
									*Baseline*: Treatment naïve group
									I: 191/mm3; C: 216/ mm3
									Treatment experienced
									I: 286/mm3; C: 348/ mm3.
									*End line*: Treatment naïve group
									I: 308/mm3; C: 298/ mm3.
									Treatment experienced group-
									I: 324/mm3; C: 356/ mm3.
									Adherence rate: I: above 98 %, C: fluctuated slightly
Thiago Martini da Costa/2012 [8]	Brazil	RCT	21	4 months	18+	SMS	Every alternative working day + Sat. + Sun. day	Self-reported adherence, pill count	Self-reported adherence
									I: remained 100 % in I group, C: 100 to 92.31 %
									Pill count
									I: 75.00 to 62.50 %.
									C: 69.23 to 46.15 %.
Lawrence Mbuagbaw/2012 [26]	Cameroon	RCT	200	6 months	21+	SMS	Weekly	Visual Analogue Scale, number of doses missed (in the week preceding interview), drug refill	No significant effect on adherence by VAS > 95 % (risk ratio1.06, 95 % CI 0.89, 1.29; *p* = 0.542; reported missed doses (RR 1.01, 95 % CI 0.87, 1.16; p.0.999) or number of pharmacy refills (mean difference 0.1, 95 % CI: 0.23, 0.43; *p* = 0.617.
									CD4 count at end of 3 months:
									C: 327/mm3 to 375/mm3
									I: 347/mm3 to 406/mm3.

**Table 3:** Key findings in the clinical area of Asthma and Hypertension

Lead Author/Year	Country	Study design	Sample size	Duration	Age group	Clinical area	Intervention	Delivery frequency	Measures of outcome	Results Control (C) versus intervention (I)
Ulla Strandbygaard/2010 [35]	Denmark	RCT	22	3 months	18–45	Asthma	SMS	Daily	Adherence to asthma treatment, lung function tests	Mean adherence rate
										I: 77.9 to 81.5 %; C: 84.2 to 70.1 %.
Keith J. Petrie/2011 [31]	New Zealand	RCT	147	4.5 months	16–45	Asthma	SMS	2 SMS/day for first 1–6 weeks, 1 SMS/day for next 7 – 12 weeks, and 3 SMS/ week for rest 13 – 18 weeks	Self-reported adherence, adherence to treatment	Self-reported adherence over all time points
										C: 43.2 %
										I: 57.8 %
										Percentage taking over 80 % of prescribed inhaler doses
										C: 23.9 %
										I: 37.7 %
E. Márquez Contreras/2004 [5]	Spain	RCT	67	6 months	18+	HTN	SMS	2 SMS / week	Adherence to drug, blood pressure measurement	Mean percentage adherence
										I: 91.1 ± 23.1 to 95.0 ± 10.4
										C: 86.2 ± 26.6 to 86.1 ± 23.4
										% of controlled hypertension at end of study-
										C: 51.5 %; I: 64.7 %.

## Results

From a total of 236 potential reseach results, 136 full texts were selected for review, of which 14 articles were included, following the inclusion criteria. These studies include 12 randomized-controlled trials, 1 quasi-experimental cohort, and 1 cross-sectional and descriptive study.

### Characteristics of reviewed studies

#### Health content area

Seven studies focused on HIV/AIDS and anti-retro viral therapy [8, 17, 24–26, 32, 33], four studies focused on diabetes management [13, 16, 34, 38], two studies examined asthma management [31, 35], and one assessed hypertension [5].

All articles focused both on secondary prevention (educate people to slow down the process of disease after being diagnosed through regular health check-up, follow physician advice regarding drug, diet, life style changes etc.) and tertiary prevention (slow down the development process of disease complications).

#### Study design and sample

Out of the fourteen studies, twelve studies employed an experimental design involving intervention and non-intervention groups [5, 8, 13, 16, 17, 25, 26, 31, 32, 34, 35, 38], one was a quasi-experimental cohort study [33], and one a cross sectional and descriptive study [24].

Regarding sample size, five studies had sample sizes below 100 [5, 8, 13, 16, 35] and out of these, two studies had a sample size of less than 25 [8, 35].

Regarding the age of participants, thirteen studies involved adults (18 years or above), and only one study focused solely on children/teenagers, e.g. 8–18 years [13].

#### Technology and mode of intervention

Out of fourteen studies, ten studies used only SMS, either short and/or long form, as an intervention. Three studies used SMS together with another intervention: one with an emergency hotline [13], one with voice call [24] and the last one used picture SMS together with an interactive voice response IVR (voice call) [33]. One study used only voice call without use of any SMS [17].

The frequency of message delivery, or voice call, differs from study to study. In two studies, SMS were delivered daily [13, 35], in three studies, SMS were delivered weekly [25, 26, 33], one study was a combination of a short SMS delivered daily and a long SMS weekly [32], in one, SMS were delivered every 3 days [34], in one, 2 SMS were delivered weekly [5], in one, 4 SMS were delivered weekly [16], and in one, 1 SMS was delivered every 4 weeks [24]. Three studies had a different system: one delivered 2 SMS weekly for the first 1-6 weeks and daily SMS for the next 7-12 weeks and 3 SMS weekly for the remaining 13-18 weeks [31]; one delivered SMS on Saturday and Sunday and every alternating working day -30 min before the last scheduled time of medicine intake [8] and one delivered SMS only if the patient didn’t open the medication dispenser [38]. In one study, patients were contacted only through a voice call once in every 2 weeks [17].

For all studies, SMS were sent in local languages or patients’ preferred languages for better access and understanding.

The duration of these studies ranged from 3 to 12 months. Six studies lasted from 3-4 months [5, 8, 16, 17, 31, 35], four lasted between 6-7 months [24, 26, 33, 38] and four studies lasted for 12 months [13, 25, 32, 34].

#### Content of the tools (message or voice call)

The content of the message varied in each intervention. Mostly, the messages consisted of simple reminders to take medicine, follow physician’s advice on diet or other life style changes activities and attend clinic/hospital on time, but a few messages were customized according to the patients’ clinical needs. Vervloet et al. [38], Pop-Eleches et al. [32], da Costa et al. [8], Strandbygaard et al. [35], and Mbuagbaw et al. [26] discussed about very precise SMS reminders of only taking pills. Shetty et al. [34], Goodarzi et al. [16], Petrie et al. [31] and Contreras et al. [5] reported messages comprised of varied instructions on medical nutrition therapy, physical activity, reminders on drug prescription and clinical visits, and healthy living habits. But not all messages were passively received, some messages provided information/prompts and required interaction from the patients. In Lester et al. [25] survey patients had to respond to the message and the clinician called back if the patient had any problem, or failed to respond to the SMS within 48 h. In Rodrigues et al. [33], patients had to answer a question about whether they had taken medicines. In some studies, patients were contacted by interactive voice calls. Kunutsor et al. [24] reported contacting the patients by voice calls or SMS in case of missed clinical appointment, as a reminder, and reasons for missed appointments were also recorded. Huang et al. [17] showed that patients were getting calls for attending scheduled visits and were welcome to ask questions concerning treatment and other health related issues. Mbuagbaw et al. [26] reported adding a contact phone number with the SMS, on which patients could call in case of emergency or any other concern.

#### Publication source

The studies about HIV/AIDS, diabetes and asthma were mainly published in the journals focusing on these specific clinical areas, while the others were published in more broadly themed journals. One article was published in an international medical informatics journal.

#### Location of research

According to the World Bank list of economics, five studies were conducted in high income countries (Denmark, Netherlands, New Zealand, Scotland, Spain), two in upper middle income countries (Brazil, Iran), four in lower middle income countries (China, Cameroon, two in India) and three studies in low income countries (two in Kenya, Uganda) [39].

### Studies measured adherence and outcome of care

Out of fourteen studies, seven studies reported only on adherence to treatment, one study focused on the clinical outcome, five studies reported to both adherence and clinical outcome and one study reported on clinical outcome together with knowledge, practice, attitude and self efficacy.

#### Adherence to treatment and health care behaviour

In the reviewed articles, the main concept of measuring adherence was taking medicine according to physician's advice. One study also used the concept of self efficacy, knowledge, attitude, practice and QoL. The tools used for measuring adherence were drug refill, pill count, and self report.

#### HIV/AIDS

Pop-Eleches et al. [32] reported that 53 % of the intervention group patients achieved adherence of at least 90 % during the intervention period of the study, compared to 40 % of participants in the control group (*p* = 0.03). Lester et al. [25] showed that adherence to ART was reported by 61.5 % in the intervention group compared to 49.81 % in the control group (relative risk for non-adherence 0 · 81, 95 % CI 0 · 69–0 · 94; *p* = 0 · 006). Self-reported adherence remained significantly better in the intervention group than the control group (odds ratio 0 · 57, 95 % CI 0 · 40–0 · 83; *p* = 0 · 0028). According to Rodrigues et al. [33] adherence increased from 85 to 94 % after 12 months of intervention, using both interactive voice call and picture message. Kunutsor et al. [24] reported mean adherence (95 % CI) 96.3 % and 98.4 %, before and after mobile phone intervention (voice call and SMS) respectively. According to Huang et al. [17], follow up rate increased from 91.3 to 95.7 % in the intervention group, whereas it showed a decreasing trend from 97.9  to 93.6 % in the non-intervention group. da Costa et al. [8] also showed that self-reported adherence remained at 100.00 % in the intervention group and decreased from 100.00 % to 92.31 % in the control group. In contrast, the pill counting method showed a decrease in both groups from the baseline to end-line, but decreased more in the control group in comparison to the intervention group (control group: from 69.23 to 46.15 % and intervention group: from 75.00 to 62.50 %). Mbuagbaw et al. [26] showed no significant effect on adherence (>95 %, risk ratio 1.06, 95 % confidence interval; *p* = 0.542) in the intervention group by VAS (Visual Analogue Scale is a psychometric response scale which is used in questionnaires as a measurement instrument for subjective characteristics or attitudes that cannot be measured directly) or self-reported missed doses (RR 1.01, 95 % CI 0.87, 1.16; p.0.999) at the end of the 6 month intervention. The mean number of pharmacy refills was also not different between groups (mean difference 0.1 95 % CI - 0.23, 0.43; *p* = 0.617).

#### Diabetes

According to Shetty et al. [34], adherence to dietary prescriptions decreased in both groups (control group: 54.5 to 52 %, intervention group: 60.3 to 58.4 %). In the same study, adherence to the physical activity advice in the control group were 47% and 52 % at the baseline and end-line, respectively. In the intervention group, the adherence improved marginally from 47 to 56 %, which was not statistically significant. Franklin et al. [13] showed an improved self-reported adherence score (Conventional Insulin Therapy alone 70.4 ± 20.0, CIT with SMS 77.2 ± 16.1, 95 % CI +0.4, +17.4, *P* = 0.042). Vervloet et al. [38] reported that patients who received SMS reminders took significantly more doses within predefined windows of time than patients receiving no reminders: 50 % vs. 39 % within a 1-h window (*p* = 0.003) up to 81 % vs. 70 % within a 4-h window (*p* = 0.007). Reminded patients tended to miss doses less frequently than patients not reminded (15 % vs. 19 %, *p* = 0.065), but days without dosing were not significantly different between groups. Goodarzi et al. [16] showed that knowledge improved from 7.97 to 10.83 %, practice from 3.72 to 4.93 %, and self-efficacy (SE) from 15.34 to 17.02 % in the intervention group, whereas there was no significant improvement found in the control group (knowledge: from 8.05 to 8.68 %, practice: from 3.86 to 4.26 %, SE: from 15.86 to 15.31 %) according to the KAP survey.

#### Asthma

According to Strandbygaard et al. [35], the absolute difference in mean adherence rate between control and intervention groups was 17.8 %, [95 % CI (3.2 - 32.3 %), *p* = 0.019]. Similarly, Petrie et al. [31] showed a significant improvement in adherence over the follow-up period in the intervention group, compared to the control group, with a relative average increase in adherence of 10 % (*p* < .001). The percentage of taking over 80 % of prescribed inhaler doses was 23.9 % in the control group compared to 37.7 % in the intervention group (*p* < .05).

#### Hypertension

According to Contreras et al. [5] the mean (±SD) adherence in the intervention group was 91.1 ± 23.1, 91.5 ± 12.0 and 95.0 ± 10.4 at 1^st^, 3^rd^ and 6^th^ months, respectively, whereas in the control group it was 86.2 ± 26.6, 87.6 ± 20.1, and 86.1 ± 23.4 (*p* = NS).

### Clinical improvement

Seven out of fourteen studies reported significant changes in the clinical outcomes as a result of voice call or SMS being sent through a mobile phone [5, 13, 16, 17, 25, 31, 34]. The remaining seven studies only measured adherence and did not look at the clinical outcomes.

#### HIV/AIDS

Out of seven studies focused on antiretroviral therapy, only three [17, 25, 26] measured clinical outcomes at the end of the intervention, either as a primary outcome or as a secondary outcome measure of adherence.

According to Lester et al. [25], plasma viral loads were reported 60.4 % in the intervention group and 48.3 % in the control group at end-line, but the intention-to-treat analysis showed weak evidence of improved suppression of viral load in the SMS group compared with the control group (OR 0.71, 95 % CI 0.50–1.01; *p* = 0.058).

Huang et al. [17] reported significant improvement in clinical outcomes (CD4 count) in the intervention group from 286/mm^3^ to 324/mm^3^, whereas in the control group they reported an increase from 348/ mm^3^ to 356/ mm^3^ within the patient group who already experienced anti-retro viral treatment. They also showed an increased CD4 count in the intervention group, from 191/ mm^3^ to 308/ mm^3^, with the treatment-naïve group patients after 3 months of intervention and 216/ mm^3^ to 298/ mm^3^ in the control group. In contrast to the above reports, Huang et al. [17], showed no statistically significant difference in mean CD4 count, weight change, WHO clinical staging, and opportunistic infections between the intervention and control groups among both treatment-naïve and treatment-experienced patients. Among treatment-naïve patients, mean QoL scores at baseline among the intervention and the control groups were the same. However, the scores after 3 months were significantly higher among patients who received a mobile call intervention in the area of physical well-being, level of independence, environment, and spiritual/religious/personal believes. Mbuagbaw et al. [26] showed an evidence of improved CD4 count at the end of 3 months from 347/mm^3^ to 406/mm^3^ in the intervention group and 327/mm^3^ to 375/mm^3^ in the control group. But no data was reported on the CD4 count at the end of the intervention of 6 months.

### Diabetes

Three studies reported on clinical outcome; 2 on type 2 [13, 16] and one on type 1 Diabetes [34].

Shetty et al. [34] reported significant improvement in the mean Fasting Plasma Glucose (FPG) level (185 + 57 mg/dl to 166+ 54, *p* < 0.002) and 2 h (PG 263 + 84 mg/dl to 220 + 67, *p* <0.002) in the intervention group at the end of one year of intervention. No significant difference in the mean HbA1C value was found in any of the groups. Serum TC (Total Cholesterol) decreased significantly in both groups (Control: 175+ 47 mg/dl to 164+ 38 mg/dl, *p* < 0.03 and intervention: 179+ 42 mg/dl to 164+ 31 mg/dl, *p* < 0.03). According to Franklin et al. [13], there was no change in HbA1c levels in patients on conventional therapy with or without voice call (10.3 ± 1.7 vs. 10.1 ± 1.7 %), but it improved in patients randomized to intensive insulin therapy plus voice call (9.2 ± 2.2 %, 95 % CI −1.9, −0.5, *p* < 0.001). The study also reported no change in mean glycaemic control in patients with Conventional Insulin Therapy (CIT alone 10.3 ± 1.7 %, CIT plus Sweet Talk 10.1 ± 1.7 %), but it improved in patients allocated to intensive insulin therapy plus Sweet Talk (9.2 ± 2.2 %, 95 % CI -1.9, - 0.5, *p* < 0.001). Goodarzi et al. [16] showed that Hb1Ac levels improved significantly in the intervention group after 3 months of intervention (from 7.91 to 7.02 %); however it also showed improvement in the non-intervention group (from 7.83 to 7.48 %). The study also reported significant improvement in other clinical outcomes: LDL (Low- density Lipoprotein) from 97.88 mg/dl to 87.93 mg/dl, Triglyceride from 179.72 mg/dl to 160.16 mg/dl, and Total Cholesterol from 180.88 mg/dl to 165.95 mg/dl.

#### Asthma

 Strandbygaard et al. [35] reported clinical outcomes for Asthma. They showed overall improvement in Exhaled Nitric Oxide (*p* < 0.001), Airway Responsiveness (*p* < 0.001) and Forced Expiratory Volume (FEV1) (*p* = 0.015), though the changes in these parameters were not significantly different in any of the groups.

#### Hypertension

Contreras, et al. [5] reported a significantly higher proportion of control of hypertension among the patients in the intervention group, (64.7 % CI, 48.6 % to 80.8 %) compared to control group (51.5 % CI, 34.4 % to 68.6 %). Among the intervention group, mean systolic blood pressure at initial level, 1^st^ month, 3^rd^ month and 6^th^ month was 158.5 ± 13.9, 143.6 ± 14.7, 141.8 ± 14.1, and 139.4 ± 13.1 mm Hg, respectively, whereas in the control group the corresponding values were 162.1 ± 13.9, 145.7 ± 11.8, 143.8 ± 13.7, and 138.3 ± 9.5 mm Hg (*p* = NS). In the same study, the mean diastolic blood pressure in the intervention group was 95.6 ± 7.9, 86.94 ± 9.8, 84.87 ± 10.1, and 84.94 ± 10.4 mm Hg, respectively, and in the control group was 95.4 ± 6.8, 86.0 ± 7.0, 85.1 ± 6.8, and 83.1 ± 5.6 mm Hg, respectively, (*p* = NS). Mean decrease in systolic blood pressure in the intervention group was 19.1 ± 14.4 mm Hg and in diastolic blood pressure, it was 10.6 ± 7.9 mm Hg. On the other hand, the mean decrease in systolic blood pressure in the control group was 23.7 ± 13.1 mm Hg and in case of diastolic blood pressure, it was 12.3 ± 7.5 mm Hg.

## Discussion

This review identified 14 health behaviour studies evaluating the effectiveness of mobile SMS and/or voice SMS or MMS, individually or in combination with other intervention modalities, such as interactive voice call or emergency hotline. This review excluded the articles which combined m-Health intervention with other web based components such as e-mail, skype, video conferencing, bluetooth and telecommunication, to find out clear evidence if m-Health alone could have an impact on the adherence and clinical outcomes, as mobile technology is more widely accessible and affordable, especially in LMICs.

There are several limitations identified in systematic review and meta-analysis, like publication bias, heterogenicity across the primary study, lack of scope of comparability and language. [1, 10]. Publication bias is a potential threat for all areas of research, including systematic review. A comprehensive search of available literature may reduce the possibility of publication bias, which occurs when studies showing positive impact of interventions are more likely to be published and cited [14]. Pre-selection of language is another potential limitation of a review paper. Excluding non-English studies may change the result of the review, as it may already exclude some important articles published in non-English journals. Including only peer reviewed articles increases the likelihood of missing a chance to access some important interventions as well. At the same time, the peer review system enhances the quality of a paper and it can be assumed that papers not published in the peer review system don’t have the same quality. Heterogenicity in the primary study was also listed as a limitation [41]. In this systematic review, twelve out of the fourteen studies were Randomized Control Trials, so the designs were not heterogenous. Despite that, we observed some differences in terms of sample size, process of intervention and tools used, which created some difficulties in drawing a conclusion about the effect of the interventions.

Five studies out of fourteen had a sample size below hundred [5, 8, 13, 16, 35] and specifically, two studies had a sample size less than 25 [8, 35] which raises the question of representativeness of the study population. da Costa et al. [8] stated that the main reason for not getting the desired sample size, was that they could not find the women who possessed mobile phones. Strandbygaard et al. [35] stated that only 26 patients were eligible for randomization for the study and, out of them, 22 patients completed the study. The questions of sample size and statistical validity of the studies are illustrative of the problems encountered with these types of interventions. Recruiting people may be difficult and/or create biase, in particular for some specific groups: women or rural inhabitants or illiterate persons who may not possess a mobile phone or may not be able to read the messages and take part in the intervention [4, 7, 21, 22, 42]. There is also a question of retaining people in the intervention when a mobile is lost/broken and/or the number changes, as is common in LMICs. According to Pop-Eleches et al. [32], 69 participants lost their phones and 51 changed phone numbers during one year course of the study. Though the process of intervention was well described in most of the articles reviewed, but the strains and/or barriers to the interventions were not well documented. Most of the articles did not mention anything about the strains or barriers to the intervention or patient drop out and/or discontinuity.

Regarding the tools used in the reviewed articles, SMS, either in short or long form, was used as the main tool in thirteen out of fourteen interventions. Voice call and emergency hot line were used as a tool in some interventions, together with SMS. But the risk of bias exists in cases using only SMS, as it targets a comparatively young and literate group of people in the intervention, rather than illiterate and older people [4, 7, 42]. Young and literate people are more likely to adopt modern technologies than others, and inclusion criteria in some of the studies were having a mobile phone or being literate and/or able to read SMS. SMS subscribers were significantly more likely to report intended or actual behaviour change (91 %) than voice subscribers, who have a lower literacy rate and/or no access to a personal mobile phone (voice call 56 %, hotline 66 %). The recall rate of last message (comprehension) was almost the same in SMS subscribers and hotline users (SMS 75 %, hotline 76 %), but slightly lower in voice message subscribers (63 %) [7]. For the management of chronic illness, a study showed that 98 % of respondents preferred to receive medication reminders via mobile phones; among those who preferred reminders, 89 % preferred only voice calls, 9 % preferred SMS and 2 % had no specific preference. [9]. According to Rodrigues et al. [33], a significantly higher proportion agreed that a voice call was more helpful when compared to an SMS (34 % preferred only voice call, 11 % preferred only SMS, 44 % preferred both the voice call and SMS, while 11 % had no specific preference). SMS seems to have a better impact on changing the behaviour, but at the same time people preferred a voice call. The potentially higher impact of SMS, may be due to a higher literacy rate and access to a personal mobile phone in the SMS group, compare to the voice call group. Age and sex as confounding factors have not been analysed. So, while SMS may be considered more efficient, easier to implement and cheaper, the population reached by this type of intervention has to be carefully considered and impact needs to be analysed.

Regarding the method of communication, most SMS were passively received, but some SMS included both passive and active or interactive components; which should make the intervention stronger and more reliable [25, 33].

The content of the tools used (mainly SMS) varied a lot from intervention to intervention.

Some SMS were a very simple and basic reminder to take medicine or make a hospital visit or for other self health care behavior. For example: “This is your reminder” [32], “Take good care of your health” [8], which may raise the question of the quality of the intervention. But some SMS interventions were customized according to the patients’ clinical need and literacy level, as well as with detailed behavioural and lifestyle change contents. In this systematic review, it was not possible to analyse the difference in the impact of the various content of the messages, as positive impact was found in almost all interventions. It may also be possible that, in the final analysis, an incoming message alert is already a reminder and the content and/or frequency is not that important. But, according to Tomlinson et al. [36], SMS is more likely to work if it is personally tailored, and the content are highly relevant.

Tomlinson et al. [36] also stated that a m-Health project is more likely to work if the project is followed up, has been designed for specific context and strong consideration has been given to the frequency of message delivery and the content of messages. The variability in tools, together with the content and frequency of use of the tools, made the comparison difficult. As a conclusion, positive effects on adherence and health outcomes were found in most of the studies reviewed and were short-term (3–12 months). Despite the positive results of the studies, it must be noted that the projects were all small-scale and success of similar large-scale or longer effect projects is not guaranteed and still in question. [3]. Therefore, further research is needed to find out the impact of the content of the message, frequency of use, the medium uses for different behavioural interventions as well as the sustainability of large scale and long term interventions. Also, the cost benefits of m-Health interventions, compared to traditional health education, are still in question, as there is no available evidence so far.

Adherence refers to the act of following the recommendations made by the provider with respect to time, dosage, and frequency of medicine intake [6, 20]. Patients’ adherence to a recommended drug regime represents the final step in a pathway from developing symptoms to receiving curative treatment [23] and the fact that patients are not taking drugs as recommended, may be more a result that patient is not having access to affordable treatment and/or not receiving the precise instructions, rather than patient related factors of non-adherence [41]. Adherence to any treatment regimen is mostly inversely proportional to the duration of treatment and the frequency of dosing. Therefore, it is more important for chronic diseases to have a strong follow up mechanism and strategy to improve patient adherence to the treatment regimen. There was a wide range of discrepancies in the definition, measurement of adherence, tools used, content and frequency of tool use, as well as in clinical areas and the country context. A patient may not fully follow the treatment regimen or advice as prescribed, but still may receive adequate treatment or follow some of the lifestyle change advice, which, at the end, may improve clinical outcomes. An 80 % compliance level for medicine intake is not enough as a measurement, as the form of drug intake may be more important than the level of compliance. For example, missing one complete week antihypertensive drug, might result in high blood pressure, with the risk of stroke and heart failure. But the same number of missed doses over a period of three-months would have no measurable effect on blood pressure control [30]. Stronger definitions of adherence in terms of dose, duration and frequency would contribute considerably in comparing the results of different studies, which is lacking in this review. Many reviews suggest that biologic assays are the most accurate measure of patient adherence, followed by pill counts, with self-report being the least accurate. However, there is no single mesure of adherence which can be recognized as the most reliable and accurate. The main problem in identifying non-compliant patients is the unreliability of many of the measures used for assessing compliance, as there are many factors; psychological, social and demographic, drug and doctor factors, which may influence the adherence at various levels [11]. Therefore, it is better to combine measures to calculate adherence and not rely on only one measure, which may produce bias or an imperfect result.

Irrespective of the differences and questions which may arise regarding methodology (design, tool, duration) and country context, eleven out of fourteen studies reported significant improvement on adherence and clinical outcomes in the intervention groups, in comparison to the control group, at the end of the intervention.

There is evidence in the published articles that m-Health works, but it is not clear what works and how it does work. Tomlinson et al. [36] stated that after completion of many pilot studies, there is almost nothing about the likely uptake, best strategies for engagement, efficacy, or effectiveness of these initiatives or information about the cost of the interventions. Flay and colleagues [12] adapted a standards model, published by the Society for Prevention Research, which was developed to guide policy, research, and practice and to provide a framework for sufficient evidence. But there are currently no m-Health interventions that meet these standards for scale up, despite numerous calls to scale up [36].

Therefore, qualitative research is very strongly recommended to determine in which conditions m-Health works, and if not, why. A process evaluation of the interventions is highly also recommended. Regarding the evaluation of impact, a more scientifically sound research design with a standard definition of adherence, clear specification of sample, and sample size, appropriate tools, effective outcome measures on health and health behaviour are recommended. In health behavioural research, testing of psychological concepts is also recommended.

As there was no cost data generated in the articles reviewed, the researchers can not make any meaningful statement about the cost-benefit aspects of the interventions. The evaluations of m-Health interventions mainly focused on feasibility, rather than impact and cost-effectiveness, which makes it difficult to draw conclusions about benefits and, in particular, additional benefits when compared to traditional interventions.

Limited funding opportunities for long-term interventions and scope of scale up at large scale threaten the sustainability of the m-Health projects at moment [37]. Most m-Health interventions considered successful in LMICs, are implemented and/or funded by the non-governmental organisations (NGOs) and not integrated into the mainstream  national level public health services [27]. Therefore, more development in the area of cost-benefit analysis research, together with the sustainability issue associated with m-Health, is required and recommended before drawing a concrete conclusion on its economic effect, in order to suggest policy advice for further decision and implementation.

## Conclusion

There is always a positive impact on adherence of disease management, as well as in health outcomes in almost all reviewed articles. But the variability in the type of intervention (tools and the use of tools) as well as in the lack of information in most of the articles about the details of the process of intervention and the concept of behavioural change, makes the conclusion difficult. Additionally, the methods used to measure the impact, as well as of the design, sample, and outcome measured (adherence measured and clinical outcomes), are also different. Moreover, the low amount of literature published on the topic also reduces the power of conclusion. However, the cost-effectiveness and sustainability issues of m-Health are still in question, especially in LMICs. But there is potential in implementing m-Health with the advancement of new technologies and a wide range of mobile network coverage; thereby offering the potential to cover the population who need long term treatment due to chronic diseases/conditions, irrespective of SES, age, and sex, and therefore make m-Health an important part of health sector.

### Availability of data and materials

Not applicable.

## Abbreviations

AIDS: acquired immuno deficiency syndrome
ART: anti-retro viral therapy
CD: communicable disease
CD4: cluster of differentiation 4
CI: confidence interval
CIT: conventional insulin therapy
DM: diabetes mellitus
FEV: force expiratory volume
FPG: fasting plasma glucose
GPRS: general pocket radio service
HbA1c: glycosylated hemoglobin
HIV: human immunodeficiency virus
HTN: hypertension
IVR: interactive voice response
KAP: knowledge, attitude and practice
LDL: low density lipoprotein
LMIC: low and middle income country
m-Health: mobile health
NCD: non-communicable disease
PDA: personal digital assistant
PG: plasma glucose
QoL: quality of life
RCT: randomised control trial
RNA: ribonucleic acid
RR: relative risk
SE: Self efficacy
SES: socio-economic status
SMS: short message service
TC: total serum cholesterol
VAS: visual analogue scale
WHO: World Health Organization
